# Medication Management Among Older Adults With Multiple Chronic Conditions: What Role Do Caregivers Play?

**DOI:** 10.1093/geroni/igaa057.1394

**Published:** 2020-12-16

**Authors:** Rachel O’Conor, Morgan Eifler, Andrea Russell, Lauren Opsasnick, Julia Yoshino Benavente, Laura Curtis, Michael Wolf

**Affiliations:** Northwestern University, Chicago, Illinois, United States

## Abstract

Many older adults manage multiple chronic conditions (MCC) that require adherence to complex medication regimens. Few studies have investigated the degree to which caregivers support medication-related behaviors. We conducted semi-structured qualitative interviews with 25 caregivers of older adults with MCC to characterize caregiver medication assistance. Two coders used content and constant comparative analysis to analyze transcripts. The mean age of caregivers was 61 years; the majority were female (68%) and identified as non-white (Black, 52%; Hispanic, 8%). Caregivers were predominantly spouses (n=10), or children (n=11). Older adults were on average 73 years old, managing 5 chronic conditions and prescribed 7 medications. Caregivers acknowledged the importance of medications to the older adult’s health, but their involvement in daily medication management was limited. Some caregivers preferred that the older adult continue these tasks to maintain autonomy, especially when caring for older adults who valued maintaining independence. Caregivers assumed medication responsibilities after older adults experienced sudden changes in health or upon observing non-adherence (e.g. full pill bottles). Older adults with higher medication burden (12+ medicines) adopted inefficient, cumbersome medication management practices; caregivers suggested simplified strategies, but the older adults refused to adopt recommended strategies. To combat resistance from the older adult, caregivers disguised assistance and deployed workaround strategies to monitor medication-taking behaviors. These findings suggest older adults and caregivers share a value of promoting independence of medication management, up until safety is seriously questioned. Additionally, there is a breakdown in communication at the time when older adults may benefit from increased caregiver involvement.

